# Developmental delays and psychiatric diagnoses are elevated in offspring staying in prisons with their mothers

**DOI:** 10.1038/s41598-018-20263-x

**Published:** 2018-01-30

**Authors:** Meryem Ozlem Kutuk, Ebru Altintas, Ali Evren Tufan, Gulen Guler, Betul Aslan, Nurgul Aytan, Ozgur Kutuk

**Affiliations:** 1 Department of Child and Adolescent Psychiatry, Baskent University School of Medicine, Adana Dr. Turgut Noyan Medical and Research Center, Adana, Turkey; 2 Department of Psychiatry, Baskent University School of Medicine, Adana Dr. Turgut Noyan Medical and Research Center, Adana, Turkey; 3 Department of Child and Adolescent Psychiatry, Abant Izzet Baysal University, School of Medicine, Bolu, Turkey; 4 Elazig Mental Health Hospital, Department of Child and Adolescent Psychiatry, Elazig, Turkey; 5 Department of Child Neurology, Baskent University, School of Medicine, Adana, Turkey; 6 Department of Neurology, Boston University School of Medicine, Boston, USA; 7 Department of Medical Genetics, Baskent University School of Medicine, Adana Dr. Turgut Noyan Medical and Research Center, Adana, Turkey

## Abstract

The aim of the study was to describe the sociodemographic and clinical features of the mothers and their offspring staying with them in prison. The study was planned as a cross-sectional, single-center study of mothers residing in Tarsus Closed Women’s Prison of Turkish Ministry of Justice along with their 0 to 6 years old offspring. Mothers were evaluated via Structured Clinical Interview for DSM-IV Axis I Disorders. A psychologist blind to maternal evaluations applied the Denver Developmental Screening Test II (DII-DST). Children/mothers were also evaluated by a child and adolescent psychiatrist via K-SADS-PL. Twenty-four mothers with a mean age of 29.3 years were included. Most common diagnoses in mothers were nicotine abuse (n = 17, 70.8%), specific phobia (n = 8, 33.3%), alcohol abuse (n = 7, 29.2%) and substance abuse (n = 5, 20.8%). Twenty-six children (53.9% female) were living with their mothers in prison, and the mean age of those was 26.3 months. Results of the D-II-DST were abnormal in 33.3% of the children. Most common diagnoses in children were adjustment disorder (n = 7, 26.9%) separation anxiety disorder (n = 3, 11.5%) and conduct disorder (n = 2, 7.7%). A multi-center study is necessary to reach that neglected/under-served population and address the inter-generational transmission of abuse, neglect, and psychopathology.

## Introduction

“Conviction” and “imprisonment” are important stress factors for psychopathology. Those experiences involve separation from friends, relatives, and acquaintances, loss of freedom, self-esteem, and privacy. Life in prison brings with its restrictions, lack of control and choice^1,2^. Previous studies carried out in various countries reported that psychiatric problems were more common in prisoners compared to the community^3–6^. The prevalence of psychopathology varied between 43.0% from Australia to 94.0% from Canada. The prevalence in Turkish population samples was in between those rates (i.e. 67.2%)^3–7^. Female prison inmates may be especially prone to develop psychopathology^7^. Motherhood is an important experience in the life of females, and the experience of being an imprisoned mother can be especially traumatic^8,9^. Conviction and imprisonment interfere in various ways with motherhood experience such as limited communication with offspring and relatives, abrupt visits with long intervals, giving birth and raising children in prison, etc^8,9^.

According to official data 6287 Turkish women inmates (3.7% of total) were serving prison sentences in 2015 and current prison policies allows mothers with children up to 6 years old to serve sentences together. Approximately five thousand of those inmates are estimated to have children up to 18 years old (Mandiraci^10^). A recent report found that 668 children through the country were in prisons with their mothers (Anonymous^11^). This option is afforded to all incarcerated women with children and there is no legal limit to the number of children who can stay with their mothers. Once the children are 6 years old they were sent to the parent outside of prison or grandparents/ other relatives pending on the familial situation. Although all women inmates are offered free academic and vocational courses during their sentences children received relatively little attention. Prison kindergartens and opportunities to foster development are limited (Mandiraci^10^).

Despite the importance of studying inmate populations, especially the vulnerable ones like women and children and adolescents for psychopathology from a public health perspective, research on those are scarce. Young children living in prisons along with their mothers may be especially prone to psychopathology. Therefore, this study was conducted to evaluate the socio-demographic and clinical features of the mothers and their infant to −6 years old offspring staying with them in prison. In a literature review, to our knowledge, there was no previous study evaluating both mental status and development of children who live with their mothers in prison.

## Materials and Methods

### Study Center and Participants

The study was planned as a cross-sectional, single-center study of mothers residing in Tarsus Closed Women’s Prison of Turkish Ministry of Justice along with their 0 to 6 years old offspring. The study protocol was approved by Baskent University Institutional Review Board and Ethics Committee (Project no: KA16/221) and Turkish Ministry of Justice. Informed consent was obtained from all participants and/or their legal guardians and the study was conducted in accordance with the principles set forth in Declaration of Helsinki and National Code of Clinical Research. The criteria for inclusion were having infant to 6 years old offspring in prison, having sufficient Turkish fluency, normal level of intellectual functioning. Exclusion criteria were a lack of Turkish fluency and sub-average intellectual functioning. At the time of the study, the prison housed 309 inmates of which 27 had infant to 6 years old offspring staying in prison together. One was not fluent in Turkish and none were judged to have sub-average intellectual functioning rendering clinical evaluations ineffective. Two mothers refused to provide Informed Consent for participation leading to a final sample of 24 mothers with 26 children in prison.

### Instruments

#### Prison Experience and Socio-demographic data Evaluation Form

This form was developed by the researchers and aimed to evaluate the socio-demographic features of mothers and their offspring. The form included information on mental disorders experienced both after and prior to imprisonment, any treatments received, attempts at self-harm and/or suicide, smoking and use of alcohol/illicit substances, family history of psychopathology, reasons for imprisonment, duration of imprisonment and prior experiences of imprisonment.

#### Structured Clinical Interview for DSM-IV Axis I Disorders (SCID-I)

This is a structured, clinical interview to evaluate DSM-IV Axis I disorders involving six modules and diagnostic criteria of 38 diagnoses. It was developed by First and colleagues^12^ and adapted to Turkish in 1999. The Turkish form was demonstrated to have adequate reliability and validity^13^.

#### Beck Depression Inventory (BDI)

This 21- item self-report inventory was developed by Beck *et al*. to evaluate levels of subjectively reported depressive symptoms^14^. Reliability and validity of the Turkish version were established by His *et al*.^15^. Each item is assessed on a 4-point Likert-type scale, and the cut-off for clinically significant depressive symptoms in Turkish version was reported to be 17^16^.

#### Beck Anxiety Inventory (BAI)

This 21-item self-report of anxiety symptoms is also graded on a 4-point Likert-type scale with higher scores denoting elevated anxiety levels. It was developed by Beck and colleagues^17^ and reliability and validity of the Turkish version were reported by Ulusoy^18^.

#### Multidimensional Scale of Perceived Social Support (MDSPS)

This 12-item, 7-point Likert-type self-report scale with scores ranging from 12 to 84 was developed to evaluate subjective levels of social support by Zimet and colleagues^19^. The scores are deemed to correlate with levels of perceived social support. Moreover, reliability and validity of the Turkish version were reported by Eker and Arkar^20^.

### Childhood Trauma Screening Questionnaire- 53 item version (CTSQ-53)

This self- report scale for assessment of retrospective recollections of childhood traumatic experiences and neglect was initially developed as consisting of 70 items^21^ with later modifications leading to 53 and 28 item versions. All forms are 5-point Likert-type scales with subscales for emotional, physical and sexual abuse during childhood and physical and emotional neglect. The Turkish versions were found to be reliable and valid previously.22Here CTSQ-53 is used.

### Denver-II- Developmental Screening Test (D-II-DST)

This screening test was initially developed in 1967 for developmental screening of 0 to 6 years old children. Multiple translations of the original exist and all demonstrated cross-cultural reliability and validity. The original test was revised in 1990, forming the Denver- II- Developmental Screening Test and reliability and validity of the Turkish version was established by Anlar and Yalaz^22^.

### Kiddie-Schedule for Affective Disorders and Schizophrenia for School-Age Children-Present and Lifetime Version-Turkish Version (K-SADS-PL-T)

K-SADS is a semi-structured interview to evaluate both present and lifetime pediatric psychiatric diagnoses according to DSM-IV-TR criteria^23^. The Turkish version was found to be reliable and valid previously^24^.

### Study procedure

In this study mothers imprisoned with their 0 to 6 years, old offspring were evaluated via SCID-I. They later completed forms for BDI, BAI, MDSPS, CTSQ-53 for themselves (with help from the study team for the illiterate). Prison Experience and Socio-demographic data Evaluation Form was completed by mothers and the study clinicians. A psychologist blind to maternal evaluations applied the D-II-DST by interviewing mothers and children. Children and mothers were also evaluated by a child psychiatrist via K-SADS- PL to determine psychopathology. The main aims of the study were to describe the socio-demographic and clinical features of the mothers and their infant-6 years old offspring.

### Statistical analyses

The data were analyzed by Statistical Package for Social Sciences (SPSS), Version 22.0. Descriptive analyses were used to summarize data. Nominal and ordinal data were summarized as frequencies while metric data were summarized either as means and standard deviations or medians and inter-quartile ranges depending on outliers/ normality. All data generated or analyzed during this study are included in this published article.

## Results

### Socio-demographic, clinical and psychometric results of mothers

Twenty-four mothers with a mean age of 29.3 (S.D. = 6.5) years were included in this study. The median period of incarceration was 365.0 days (IQR = 625.0). The mothers’ socio-demographic and clinical features were illustrated in Table 1.

**Table 1:** Sociodemographic and clinical features of mothers’ with infants to 6 years of offspring imprisoned at the Tarsus Closed Womens’ Prison.

	**Mean**	**Standard Deviation**	**Range**
Age at marriage (years)	16.9	3.4	13.0–28.0
BDI	24.7	13.6	7.0–58.0
BAI	20.9	12.1	1.0–52.0
MDSPS	48.0	19.8	12.0–84.0
CTSQ-53-Emotional abuse	28.7	14.3	12.0–55.0
CTSQ-53-Physical abuse	15.3	8.8	7.0–32.0
CTSQ-53- Sexual abuse	10.2	5.4	7.0–29.0
CTSQ-53- Emotional neglect	46.4	16.7	16.0–77.0
CTSQ-53-Physical neglect	15.7	5.9	8.0–30.0

BDI: Beck Depression Inventory, BAI: Beck Anxiety Inventory, MDSPS: Multidimensional Scale of Perceived Social Support CTSQ-53: Childhood Trauma Screening Questionnaire-53 item form.

Mean scores of mothers in CTSQ-53 were 10.9 (S.D. = 4.2, Range = 5.5–20.6) points. When previously reported cut-offs for the Turkish version of the CTSQ-53 was used^19^; all mothers scored in clinical ranges for emotional abuse, emotional neglect, and physical neglect while none scored in the clinical range for total scores.

The majority of the mothers were married and home-makers (n = 23, 95.8%). For the majority, the marriage ceremony was religious (n = 13, 54.2%) and they had other offspring living outside of prison (n = 18, 75.0%). The level of education was low with ten being illiterate (41.7%) and 2 of the mothers being barely literate (8.3%). Apart from those most of the remaining had a secondary level of education (n = 8, 33.3%). Maternal grandparents were also mostly illiterate (62.5% and 45.8% for grandmothers and grandfathers; respectively). The spouses of the women were educated mostly at the secondary (33.3%) or primary (25.0%) school levels and worked mostly as unqualified workers (62.5%). Income of the families was mostly below the poverty level (83.3%). Three of the women were imprisoned with pending trials (12.5%) while the rest received their sentences and were serving them. For those with terms to serve mean duration remaining was 5.9 years (S.D. = 6.8). The crimes were theft (62.5%), substance use/dealing (20.8%) and murder (12.5%), in decreasing frequency. Half of the women were imprisoned previously for varying durations and offenses while 41.7% reported that their spouses and 70.8% indicated that other family members were currently serving prison terms. Almost half (41.7%) of the women stated that they were receiving treatment for chronic medical conditions (mostly asthma; n = 7, 29.2%).

A third of the mothers reported contact with mental health services before their imprisonment while 9 of them (37.5%) were receiving psychiatric treatment as prison inmates at the time of the study. Evaluation with SCID-I revealed psychopathology in the majority (n = 17, 70.8%), almost half receiving no treatment (Table 2). The family history of psychopathology was elicited from 45.8% of the mothers. Self- reported domestic abuse (54.2%) and history of self-harm (45.8%) was common while self- reported sexual abuse was rarer (8.3%).

**Table 2:** Lifetime SCID-I diagnoses of mothers’ with infants to 6 years of offspring imprisoned at the Tarsus Closed Womens’ Prison.

**Diagnosis**	**N**	**Rate**
Nicotine abuse	17	70.8%
Specific phobia	8	33.3%
Alcohol abuse	7	29.2%
Substance abuse	5	20.8%
Major Depressive Disorder	3	12.5%
Conversion Disorder	3	12.5%
Obsessive Compulsive Disorder	1	4.2%
Substance dependence	1	4.2%

### Sociodemographic, clinical and psychometric results of children

The median number of children reported by mothers were 3.0 (IQR = 8.0). Most of the mothers had one child living in prison while two mothers have two of their children each, living along. Twenty-six children (53.9% female) were living with their mothers at the time of the study, and the mean age of those was 26.3 (S.D. = 16.1) months. Delivery for those children was mostly uncomplicated/vaginal and at term (75.0% and 79.2%; respectively). Motor- mental developmental milestones of children are listed in Table 3.

**Table 3:** Motor- mental developmental milestones of children infants to 6 years old living with their imprisoned mothers in Tarsus Closed Women’s Prison (all values in months).

	**Mean**	**Standard Deviation**	**Range**
Breastfeeding	8.3	8.8	0.0–36.0
Walking	10.0	6.0	9.0–24.0
Speech	13.5	10.0	7.0–36.0
Toilet training	13.1	13.5	12.0–39.0

Children were living in prison along with their mothers for a median of 195.0 days (IQR = 375.0). Fifteen of the children (57.7%) were attending the prison kindergarten. Most (80.8%) could play with their toys in the prison while 45.8% could draw pictures/use pencils. However, only 4 of the children (15.4%) had books read to them by their mothers and/or spent special time together. Results of the D-II-DST were abnormal in 33.3% of the children. Additionally, one of the children had cerebral palsy (3.9%). Half of the children received psychiatric diagnosis via semi-structured evaluation by a child psychiatrist (Table 4). None of those children was previously evaluated by child psychiatrists, and they had been given no treatment previously.

**Table 4:** Lifetime diagnoses according to K-SADS of infants to 6 years old offspring of mothers’ imprisoned at the Tarsus Closed Womens’ Prison.

**Diagnosis**	**N**	**Rate**
Adjustment disorder	7	26.9%
Separation Anxiety Disorder	3	11.5%
Conduct Disorder	2	7.7%
Attention Deficit/ Hyperactivity Disorder	1	3.9%
Enuresis Nocturna	1	3.9%

## Discussion

This single center, cross- sectional study of imprisoned mothers living with their infant to 6 years old offspring in Tarsus, Turkey aimed to evaluate their socio-demographic characteristics and clinical features of mothers and their offspring. As a result, we found that those mothers were mostly illiterate, poor and without formal education, their marriages were mostly religious, they had other family members serving/served prison times and that they have elevated rates of family psychopathology, domestic abuse, and self- harm. Motor-mental developmental milestones in offspring were within normal limits on average, although one-third showed signs of developmental delays. Imprisonment interfered with motherhood in our sample. The majority of the mothers and half of the offspring met criteria for psychopathology. Most common disorders in mothers were nicotine abuse, specific phobia and alcohol abuse while most common disorders in offspring were adjustment disorder, separation anxiety, and conduct disorder. Half of the mothers with psychopathology and all of the children with psychopathology were not receiving treatment for their problems.

Research has consistently shown that prisoners have high rates of psychiatric disorders globally^25^ although the status of imprisoned women has been relatively neglected^7–9^. Previous studies from US and UK reported rates of psychopathology among female inmates as being 70.0 to 76.0%^8,9^. Most of those studies reported that alcohol and substance abuse are relatively common with PTSD following closely^8,9^. Boşgelmez *et al*. indicated that the rate of lifetime suicide attempts/self- harm as 50.0% in her sample while that of domestic abuse was 63.3%^7^. In that study, rates for nicotine, substance, and alcohol abuse were found to be 60.0%, 13.3%, and 6.7%; respectively. Although there are other isolated studies conducted on Turkish women prison inmates, none focused on psychopathology *per se*^26,27^. All studies focusing on psychopathology among women inmates reported that despite their elevated levels, those disorders were frequently under-diagnosed and poorly treated. In our study, consistent with the literature, evaluation with SCID-I revealed psychopathology in the majority of women with almost half receiving no treatment and about one-third receiving psychiatric treatment as prison inmates at the time of the study. Rates for nicotine abuse were also similar to those previously reported.

According to Turkish Institute of Statistics; women formed 3.7% of the 173.814 people convicted/imprisoned in 2015. Also, according to those data, imprisoned women were mostly poorly educated, between 25–29 years and most common crimes committed were theft and producing/selling illicit substances^28–30^. Similar to the official statistics and previous literature, our sample of imprisoned/convicted women was also mostly uneducated and from lower socio-economic levels. The age range and committed crimes were also consistent with previous studies and reports.

Social support for prison inmates may involve friends, family, and institution and helps to protect their physical and psychological health^7^. Boşgelmez *et al*.^7^ reported that perceived social support among her sample in prison is significantly lower for female inmates and Çoban *et al*.^29^ indicated that women in prison perceived a moderate level social support. Consistent with those reports and according to MDSPS scores, the level of social support in our sample was also moderate.

The relationship between criminal offending and early childhood adverse experiences is one of the widely reported and supported tenets of developmental psychopathology. It is also valid for female offenders^30–34^. In our study, consistent with the literature all inmate mothers scored in clinical ranges for emotional abuse, emotional neglect, and physical neglect. It is also widely known that childhood adverse experiences increase the risk for developing internalizing and externalizing disorders over the lifespan^35,36^. We also found elevated rates of psychopathology in our sample. This finding may be due to limited and biased sample, recall and/or reporting bias and should be replicated with further studies.

The preschool period is a critical time for developing skills in various domains, and its effects are felt through the lifespan of the individual^37^. In children with risk factors, developmental delays are among the most frequently observed problems with a 3–25% prevalence in the first six years of life^38^. Risk factors identified in the literature that compromise children’s development and the developing brain include biological (e.g. stunting, infections, anemia, preterm birth), psychosocial (e.g., inadequate cognitive stimulation, exposure to violence, household dysfunction), socio-demographic risk factors (e.g., poverty)^39–42^ and poor mental health in mothers^43^. Childhood trauma and domestic violence in mothers themselves, as well as teenage motherhood, are also significant risks for the cognitive delay^44–46^. On the other hand; protective factors against delayed child development in at-risk samples include; daily interaction with child (i.e. reading, imitation games, singing), higher parenting self-efficacy, higher social support and community engagement^47^. Such protective factors may be especially important in imprisoned mothers^48^. Drastically, children spent imprisonment time with their mother in unacceptable and overpopulated prisons, and they become victims of the system similar to our findings. A recent report on the status of more than 1500 children of imprisoned parents from various countries of European Union found that parental imprisonment affects children independently of national affluence and conditions in prison in different ways. Those include stigmatization, separation/attachment issues, confusion/ambiguity about parents’ loss/incarceration. According to those results, mental health problems in those children are elevated, especially for those younger than 11 years^49^. Accordingly, modification in arrest/policing processes, consideration of children’s perspectives in legal proceedings and the importance of receiving information about imprisonment from parents are suggested to promote resilience^49^. Although 668 children were reported to live with their mothers in the Turkish prison system, there were no previous reports other than ours to evaluate their psychological status and development (Anonymous^11^). Therefore we can report that our results are consistent with findings from EU countries but ecological validity for other samples of children living in other prisons should be evaluated.

Some of the features of the study setting may have affected our results. A vast majority of mothers were at the low socio-economic level, had a psychiatric disorder, got married and gave birth during adolescence period, and literacy rate was quite low. Moreover, 50 inmates stayed in a ward in prison and unfortunately children had to sleep in the same bed with their mothers. This feature may have elevated rates of separation anxiety disorder in our sample. A room of the prison was reserved as kindergarten, but there was no child-friendly environment. Sometimes other women were angry with children for being noisy and swore at them. Quarrels broke out frequently inwards increasing the children’s insecurity. Fortunately, most of the children in our sample could play with their toys in the prison while almost half could draw pictures/use pencils. However, only 4 of the children (15.4%) had books read to them by their mothers and/or spent special time together. Reflecting the adverse conditions, one-third of our sample of children was delayed in developmental milestones. Half of the children received psychiatric diagnosis via semi-structured evaluation by a child psychiatrist. The most common diagnoses were adjustment disorder (especially among recent arrivals/younger children), separation anxiety and conduct disorder. A more structured and enriched environment, special interaction times between mothers and their children, developmentally informed child care, structured curricula for imprisoned mothers in child development and positive disciplinary practices, providing developmentally appropriate information to children about their surroundings and circumstances may reduce rates of psychopathology and prevent inter-generational transmission of traumatic experiences^48–50^.

## Conclusion

This single-center, cross- sectional study of mothers in prison living with infant to 6 years old offspring in Tarsus Closed Women’s Prison aimed to evaluate the mothers via semi-structured interviews for psychopathology and self-report scales for childhood trauma, perceived social support, depression and anxiety and their offspring with developmental screening tests and semi-structured interviews. We found that the majority of mothers had low levels of education, living in poverty, married early and lacked civil protection for their marriages and offspring. Self- reports of domestic abuse and suicide were high while all mothers scored in clinical ranges for emotional abuse, emotional neglect and physical neglect in self-reports. The majority had psychopathology, often unrecognized and untreated by the prison authorities. Most common diagnoses in mothers were nicotine abuse, specific phobia, alcohol and substance abuse and those with psychopathology tended to have elevated CTSQ-53 sexual abuse scores.

Children in prison were often neglected by their mothers themselves. Half of those fulfilled criteria for psychopathology while one-third were delayed in developmental screening tests. Most common diagnoses in children were adjustment disorder, separation anxiety disorder, and conduct disorder. None of the children with psychopathology were previously evaluated, and none received previous treatment. The main limitations of our study are its cross-sectional nature and dependence on mothers as sole informants. Sampling, recall and reporting bias may also have affected our results. There may also be differing motives for mothers to report problems in their offspring (i.e. to seek treatment, to have their sentences reduced and/or being granted extra privileges by the prison administration). Regardless of its limitations, this study shows that both imprisoned mothers and their preschool offspring may be under elevated risk of psychopathology and that their psychopathology may be often unrecognized. A multi-center study, preferably under the aegis of the Turkish Association of Child and Adolescent Psychiatry to evaluate mothers imprisoned with their 0 to 6 years old offspring may be necessary to reach this neglected and under-served population and address the inter-generational transmission of abuse, neglect, and psychopathology. Furthermore, we suggest opening child and mother-friendly prisons with special rooms, creches, and playgrounds. As per suggestions outlined in EU reports; modification in arrest/policing processes, consideration of children’s perspectives in legal proceedings and the importance of receiving information about imprisonment from parents may promote resilience and reduce psychopathology in offspring^49^.
